# Using emulated clinical trials to investigate the risk of being diagnosed with psychiatric ill health following the cancer diagnosis of a sibling

**DOI:** 10.1371/journal.pone.0298175

**Published:** 2024-04-18

**Authors:** Sara Kjellsson, Kristiina Rajaleid, Bitte Modin

**Affiliations:** 1 Department of Public Health Sciences, Stockholm University, Stockholm, Sweden; 2 Department of Psychology, Stress Research Institute, Stockholm University, Stockholm, Sweden; University of Botswana, BOTSWANA

## Abstract

**Background:**

The sibling bond is often the longest relationship in an individual’s life, spanning both good and bad times. Focusing on the latter, we investigated whether a cancer diagnosis in one adult sibling is predictive of psychiatric illness in the other, and if any such effect differs according the ‘sociodemographic closeness’ between the siblings in terms of sex, age, education, marital status and residence.

**Methods:**

We used hospital records to identify psychiatric diagnoses (2005–2019) in a Swedish total-population cohort born in 1953, and cancer diagnoses (2005–2017) in their full siblings. By means of emulated clinical trials, the cohort member’s risk of a diagnosis within two years following a first exposure (or non-exposure) to a sibling’s cancer was analyzed through Cox regression.

**Results:**

Exposed cohort members had a higher risk of psychiatric diagnosis than unexposed (HR = 1.15; CI: 1.08–1.23), with men displaying a higher risk (1.19; CI: 1.09–1.31) than women (HR = 1.11; CI: 1.01–1.22). Sub-analyses of the exposed group showed that women with a cancer-stricken sister had a higher risk of adverse psychiatric outcomes (HR = 1.31; CI: 1.07–1.61) than women with a cancer-stricken brother. Furthermore, unmarried cohort members ran a higher risk, both when the cancer-stricken sibling was married (HR = 2.03; CI: 1.67–2.46) and unmarried (HR = 2.61; CI: 2.16–3.15), than in cases where both siblings were married. No corresponding difference were detected for ‘closeness’ in age, education and residence.

**Conclusions:**

In line with theories of linked lives, our findings suggest that negative events in one sibling’s life tend to ‘spill over’ on the other sibling’s wellbeing, at least during the 15-year-long period leading up to retirement age.

## Background

The concept of linked lives highlights that people are embedded in networks of social relationships, and that those with whom we form social bonds influence our lives in numerous ways [1]. Siblings often provide the most long-lasting relationships in an individual’s life, with bonds upheld by shared memories and reference points as well as a network of interlocking family relationships [2]. Having siblings can positively impact socio-emotional development [3, 4], and is also related to favourable long-term outcomes such as reduced risk of poverty [5] and lower mortality [6], compared to being the only child. Moreover, a warm relationship with a sibling is positively linked to well-being [7], and siblings generally continue to be important attachment figures and sources of emotional and practical support throughout life [8]. Previous research indicates that although siblings tend to play a less central role in people’s networks during young adulthood and family-formation, they usually regain their position as important sources of social support during the later stages of people’s lives [9].

By the logic of linked lives, the tie between siblings means that they do not only share the joys, but also the hardships, of life. The research area on bereavement, for instance, shows that the loss of important family members affects health and mortality, and this is true also for the loss of a sibling [10]. Having a close relative suffering from chronic or severe illness, such as cancer [11] or mental ill health [12], can cause emotional distress as well as a commitment (or perceived obligation) to support and care for that person. This can involve practical, material and emotional burdens that may invoke strain and subsequent psychosocial distress [13].

According to Stålberg and colleagues [12], severe illness in a sibling affects the individual via emotional responses (positive and negative) and coping strategies (both distancing behavior and increased caregiving or grieving) as well as through the fear of possible heredity of disease. Although the authors refer specifically to siblings with schizophrenia, similar responses are likely to be activated also for other severe illnesses. How deeply affected one becomes by a sibling’s illness is likely to depend on the strength of the emotional bond to the sibling in question. This bond can be assumed to be stronger when siblings share similar sociodemographic characteristics such as sex [14–17], age [18],marital status and educational level [9], or if they live in close proximity to each other [9].

In this study, we emulate clinical trials to estimate the presumed ‘downside’ of linked lives, namely whether the onset of severe illness in one adult sibling tend to ‘spill over’ on the other sibling’s health. Based on medical records for a Swedish cohort born in 1953, and their siblings, we investigate whether a sibling’s cancer diagnosis increases the risk of developing a psychiatric illness over a 2-year period. The analyses cover a 15-year-long period leading up to the cohort member’s retirement age (2005–2019). Among exposed cohort members, furthermore, we examine whether any risk of psychiatric illness is higher if the siblings are ‘close’ to each other in terms of sociodemographic characteristics. We posit two hypotheses, one for the overall relationship between sibling cancer and psychiatric diagnosis (H1) and one for variation by sociodemographic closeness in the exposed group (H2) (see Table 1).

**Table 1:** Specification of research questions (RQ) and resulting hypotheses (H).

**RQ1**	**Does the onset of severe illness in one adult sibling ‘spill over’ on the other sibling’s mental health?**	
	H1	The risk of getting a psychiatric diagnosis within the next-coming two years is higher for individuals whose sibling receives a cancer diagnosis, than for individuals whose sibling(s) do not receive a cancer diagnosis.
**RQ2**	**If so, is the effect of one sibling’s severe illness on the mental health of the other sibling stronger in cases where the siblings are ‘close’ to each other in terms of sociodemographic characteristics?**	
	H2	The risk of getting a psychiatric diagnosis within two years from a sibling’s cancer diagnosis is higher when the siblings are ‘close’ to each other in terms of sex, age, educational level, marital status and place of residence.

## Method

We followed the framework for emulated clinical trials (ECT) as discussed by Hernán and Robins [19]. The idea of ECT is to use available observational data, rearranging them as ‘trials’, and applying rules for inclusion in the study that emulate the inclusion criteria that would have been used had it been a randomized control trial (the so-called *target trial*). We emulate a ‘target trial’ of the effect of a sibling’s cancer diagnosis on the risk of psychiatric diagnosis.

Eligibility criteria included being born 1953 and living in Sweden, not having a current psychiatric diagnosis, having at least one full biological sibling and no cancer diagnosis in a sibling during previous 12 months. Treatment was defined as having a sibling with a newly detected cancer. The outcome was defined as being diagnosed with affective, neurotic or stress-related psychiatric illness within a 24-month follow up period. We applied Cox proportional hazard regression and our target trial estimated the *intention-to-treat effect* [19]. For detailed description of the protocol, see Table 2.

**Table 2:** Protocol for the emulation of a target trial to estimate the risk of psychiatric illness following sibling cancer, using observational data from administrative health care records from the RELINK53-data.

Protocol	Target trial	Emulated trial
Eligibility criteria	1. Born 1953 2. Not adopted 3. Alive and living in Sweden at the start of trial 4. At least one full biological sibling alive and living in Sweden at the start of trial 5. Does not have a current diagnosis of affective, neurotic, stress-related, substance use related, sleep- or eating disorder at the start of trial 6. No sibling with a cancer diagnosis during the past 12 months 7. Has not been included in the exposed group in a previous trial 8. No missing information on variables included in analysis	Same as for the target trial. RELINK53-data contain family linkages with both biological and adoptive parents, enabling definition of non-adopted individuals and of full siblings. We defined living in Sweden as being registered as resident in a Swedish municipality. For the years 2016–2019, information on municipality of residence is not available in RELINK53-data and, for those years, individuals are defined in accordance with information from 2015 with exception for individuals with a registered emigration from Sweden after 2015 (and before start of trial). Psychiatric diagnoses registered during the past 12 months are defined as ‘current’. ICD-codes used: F10-F19, F30-F39, F40-F48, F50, F51, F55.
Treatment strategies	Exposed: Having a sibling with a newly detected cancer at baseline. Unexposed: Not having a sibling with cancer at baseline.	Same as for the target trial As a newly detected cancer we define the month of an in- or outpatient healthcare visit with a registered cancer diagnosis that has not been preceded by a previous visit with a cancer diagnosis for the past 12 months. All diagnoses in ICD-10 chapter II are used, with the exception for benign tumours (D10-D36).
Treatment assignment	Individuals are randomly assigned to a treatment strategy at baseline.	Individuals were assigned to the treatment strategy that was aligned with their data at baseline. Number of siblings alive and living in Sweden at the start of trial is adjusted in analysis to emulate random assignment.
Outcome	Diagnosed with affective, neurotic or stress-related psychiatric illness.	Same as for the target trial. Psychiatric illness defined by ICD-10 codes referring to affective, neurotic or stress-related disorders, substance use, sleep- and eating disorders: F10-F19, F30-F39, F40-F48, F50, F51, F55.
Follow-up	Starts at baseline with a 24-month follow up period. Ends at a psychiatric diagnosis, death, migration, loss to follow-up on any of the included analytical co-variates or at end of follow up. Trials in the unexposed group are further ended if a sibling is diagnosed with cancer during the follow-up time.	Same as for the target trial. Using a sequence of emulated trials starting at each of the 156 months from January 2005 to December 2017.
Causal contrast	Intention-to-treat effect.	Registered diagnosis codes of cancer can imply both suspected and detected cancer. With only registered ICD-codes we cannot distinguish between these. Thus, being an observational analogue of the intention-to-treat effect: exposure at baseline is registered but continued exposure cannot be ascertained.
Statistical analysis	Cox proportional hazard regression for the risk of being diagnosed with psychiatric ill health over a two-year period.	Same as for the target trial.

**Fig 1:**
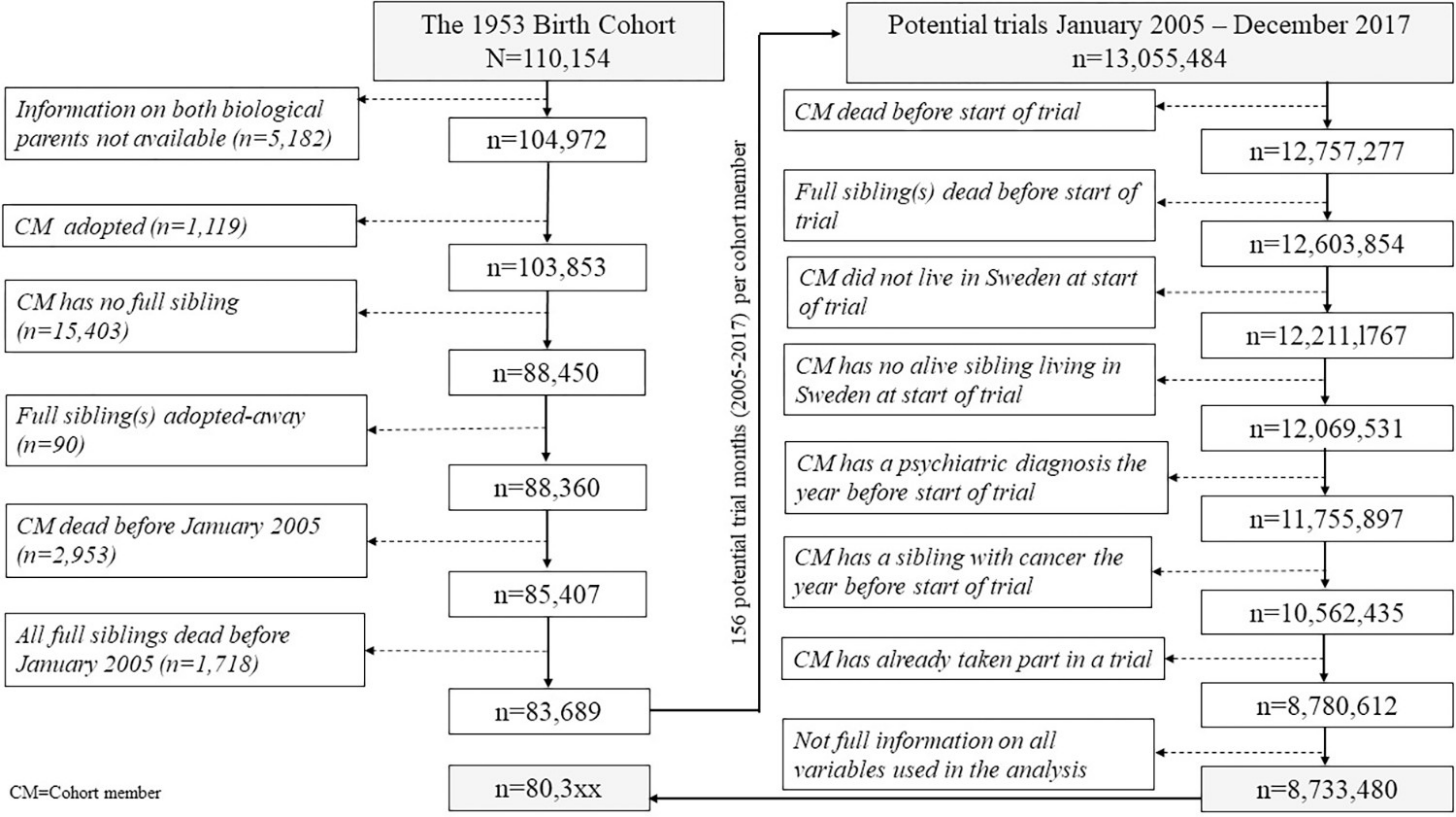
Flow chart of the exclusion procedure. From the full birth cohort of 1953 to individuals used in analyses (left side) and from potential trials to eligible trials used in analyses (right side).

We used RELINK-53 data, which is a compilation of administrative registers for a Swedish total-population cohort born in 1953 and their family members. Detailed description of this data is available in a cohort profile [20]. We included non-adopted cohort members with at least one non-adopted full biological sibling (n = 104 972) and followed them from 2005 to 2019 (between 52–66 years of age). Fig 1, left side, describes the applied exclusion criteria.

We defined each month, from January 2005 to December 2017, as the starting point for an observation period, a ‘trial’, with a follow-up of 24 months. This resulted in 156 trial-months for each eligible cohort member, with the last observation of outcome occurring in December 2019. Cohort members whose sibling received a cancer diagnosis during the month starting the trial were placed in the treatment group (hereafter: the exposed group). Unexposed cohort members (i.e., no sibling with cancer) were placed in the non-treatment group. Cohort members were included in all monthly trials where they met the eligibility criteria. They were followed from baseline to outcome (psychiatric diagnosis), and were censored at death, emigration, or (for the unexposed group) if a sibling became diagnosed with cancer during the trial period.

Overall, 8 733 480 observable trials fulfilled the eligibility criteria, corresponding to 80 281 (73%) of the initial 110 154 cohort members (Fig 1, right side).

As exposure, we used the first cancer diagnosis of a sibling that the cohort member experienced during follow-up, making sure that no such diagnosis was registered during the preceding 12 months. A cohort member, whose sibling was diagnosed with cancer, contributed one trial starting from the month of the first cancer diagnosis. Additionally, the same cohort member contributed multiple 24-month-long trials as part of the unexposed group, with each trial beginning in months when they were not exposed to their sibling’s cancer. Cohort members who never experienced a sibling’s cancer diagnosis only contributed multiple trials as members of the unexposed group.

A short description of the variables included in the analyses is given below (for details, see Table 3).

**Table 3:** Variables included in analyses.

Type	Content	Source	Coding	Variable entered
Exposure	Sibling cancer	National Patient Register	All physician contacts, as an in- or outpatient, where a diagnose of cancer is registered. ICD-10 codes, defining exposure as any diagnose from ICD-chapter II, with the exceptions for benign tumours (D10-D36).	Sibling with cancer diagnosis = 1; No sibling with cancer diagnosis = 0
Outcome	Affective, neurotic, stress-, substance use-, sleep- or eating-related disorders	National Patient Register	All physician contacts where a psychiatric diagnosis is registered referring to affective, neurotic, stress-related, substance use-related, sleep-related or eating-related disorders. ICD-10 codes used: F10-F19, F30-F39, F40-F48, F50, F51, F55. Diagnoses are registered at the date of in- or outpatient visit.	Psychiatric diagnosis = 1; No psychiatric diagnosis = 0.
Co-variates in analyses on both exposed and unexposed	Sex	The Total Population Register	Men = 1; Women = 2	Woman = 1; Man = 0.
	Age at start of trial	The Total Population Register	Year of trial—1953 (all cohort members born in 1953)	Continuous variable
	Education	Longitudinal Integration Database for Health Insurance and Labour Market Studies (LISA)	Highest attained educational level (Sun2000niva). Collapsed into 3 levels: i) lower secondary education or less, ii) upper secondary education, iii) tertiary and postgraduate education. Available annually for 2005–2015, for trials starting 2016–2017 highest education in 2015 is used.	Categorical dummy variables. Reference category = Tertiary and postgraduate education.
Co-variates in analyses on both exposed and unexposed	Marital status	Longitudinal Integration Database for Health Insurance and Labour Market Studies (LISA)	OG = Unmarried G = Married S = Divorced Ä = Widow/widower RP = Registered partner SP = Divorced partner EP = Surviving partner Collapsed into Married/registered partner (G + RP) and Unmarried/unpartnered (OG + S + Ä + SP + EP)	Married/registered partner = 1; Unmarried /unpartnered = 0.
Co-variates for sociodemographic closeness, in sub-analyses on the exposed group	Sibling sex composition	The Total Population Register	Combination of cohort member’s sex and sex of the sibling with cancer at the start of trial: i) Two brothers, ii) Two sisters, iii) Cohort member brother and sibling sister, iv) Cohort member sister and sibling brother	Categorical dummy variables. Reference category = Cohort member sister and sibling brother.
	Age difference	The Total Population Register	1953—age of sibling with cancer at the start of trial. Divided into categories: i) age difference < = 2 years, ii) sibling with cancer > 2 years older than cohort member, iii) sibling with cancer > 2 years younger than cohort member	Categorical dummy variables. Reference category = sibling > 2 years older than cohort member
	Closeness by place of residence	Longitudinal Integration Database for Health Insurance and Labour Market Studies (LISA); Source for coordinates: https://sv.wikipedia.org/wiki/Lista_%C3%B6ver_Sveriges_kommuner, accessed 2021-12-29.	Distance calculated using longitude and latitude for the residential city of each municipality. Municipality of residence is available annually between 2005–2015. For 2016–2017 information from 2015 is used, with exception for individuals with a registered move from Sweden after 2015 and before start of trial. Collapsed into categories: i) same municipality, ii) < = 200 km distance, iii) 201–500 km distance, iv) > 500 km distance	Categorical dummy variables. Reference category = Same municipality.
Co-variates for sociodemographic distance, in sub-analyses on the exposed group	Marital status composition	Longitudinal Integration Database for Health Insurance and Labour Market Studies (LISA)	Combination of cohort member’s and sibling’s marital status: i) Both married, ii) Cohort member married and sibling unmarried, iii) Cohort member unmarried and sibling married, iv) Both unmarried.	Categorical dummy variables. Reference category = Both married
	Educational level difference	Longitudinal Integration Database for Health Insurance and Labour Market Studies (LISA)	Combination of cohort member’s and sibling’s highest attained education at start of trial. Categorized as: i) Same educational level, ii) Sibling higher educational level than cohort member, iii) Cohort member higher educational level than sibling.	Categorical dummy variables. Reference category = Same educational level
Control variables	Number of siblings alive and living in Sweden at start of trial.	The Total Population Register; Longitudinal Integration Database for Health Insurance and Labour Market Studies (LISA)	Sum of siblings that are i) not registered as deceased during or prior to the trial month, ii) have a registered municipality of residence in Sweden during the year. Municipality of residence is available annually between 2005–2015. For 2016–2017 information form 2015 is used, with exception for individuals with a registered move from Sweden after 2015 and before start of trial.	Continuous variable.
	Previous psychiatric diagnoses	National Patient Register.	Physician contacts prior to start of trial, as an in- or outpatient, with diagnoses referring to affective, neurotic or stress-related illness, substance use, sleep- or eating disorders. Includes diagnoses from 1997 and onward.	Previous diagnosis = 1; No previous diagnosis = 0

Exposure. For sibling cancer diagnoses, we used information from the National Patient register and included all physician contacts, as in- or outpatients, where a diagnosis of cancer was registered. We defined exposure as having any sibling diagnosed from ICD-10 chapter II, except benign tumours (D10-D36).

Outcome. For psychiatric diagnoses, we used information from the National Patient register on conceivable diagnoses that could have been triggered by an emotional response in the cohort member following the sibling’s cancer diagnosis. We selected affective, neurotic, stress-, substance use-, sleep- and eating-related disorders (for ICD-10 codes, see Table 2). For simplicity, we will refer to this as psychiatric diagnoses.

Sociodemographic information was retrieved from the Longitudinal Integration Database for Health Insurance and Labour Market Studies (LISA). *Educational level* was divided into i) lower secondary, ii) upper secondary, and iii) tertiary. *Marital status* was categorised as married/partnered or unmarried/unpartnered. These variables were used as co-variates in the first part of the analyses. In the second part, both cohort member’s and their siblings’ information on these variables were used to define ‘sociodemographic closeness’ (see below). We further collected annual information on municipality of residence for cohort members and their siblings, which was used to calculate geographical closeness.

### Analytic strategy

The statistical method used was Cox proportional hazard regression [21] with individual-clustered standard errors. The assumption of proportional hazards was tested and met. All models were controlled for age at start of trial.

To test our first hypothesis, hazard ratios (HR) were estimated for the full sample, and for women and men separately. Models were controlled for marital/partnership status, educational level, and number of siblings. Finally, we also adjusted for previous psychiatric diagnoses (from age 44 and onwards).

In order to test our second hypothesis of the role of the ‘sociodemographic closeness’ between the cohort members (CM) and their cancer-stricken siblings, we restricted the analyses to the exposed group only. For *age*, we categorized the sibling as being i) no more than two years younger or older than CM, ii) more than two years older than CM, and iii) more than two years younger than CM. *Sibling sex composition* was categorized into: i) two brothers, ii) two sisters, iii) CM brother and sibling sister, and iv) CM sister and sibling brother. *Educational level* was defined as the sibling having i) the same, ii) lower, or iii) higher educational level than CM. *Marital status* was divided into i) both CM and sibling married, ii) CM married and sibling unmarried, iii) CM unmarried and sibling married, and vi) both CM and sibling unmarried. We also investigated the potential role of *geographical closeness* between the cohort members and their cancer-stricken siblings, based on the geographical coordinates (longitude and latitude) of their respective municipality of residency [22]. This variable takes account of whether the sibling-pair lived i) in the same municipality, ii) within 200 km distance, iii) within 201–500 km distance, or iv) with more than 500 km distance from each other. As information about municipality of residency was only available until December 2015, a sensitivity analysis was performed that excluded trials starting from January 2016 and from January 2014. For this second part of the analyses, exposed individuals each contributed with one trial (n = 34 457).

## Results

Variable distribution is presented in Table 4. Results regarding our first hypothesis are presented in Table 5. The age-adjusted baseline model shows a HR of 1.15 (CI 1.08–1.23) of receiving a psychiatric diagnosis within two years following the exposure to a sibling’s cancer diagnosis (Model 1:1). Adjustment for marital status, educational level and number of siblings (Model 2:1) only marginally affects this estimate. When previous psychiatric diagnosis is controlled for (Model 3:1), the excess risk drops somewhat. Gender separate analyses reveal a lower age-adjusted HR for exposed women (HR = 1.11, CI 1.01–1.22) than for exposed men (HR = 1.19, CI 1.09–1.31) (Models 1:2 vs. 1:3). The HR for exposed women is not affected by adjustment for co-variates (Model 2:2), but when previous psychiatric diagnoses are included (Model 3:2), the risk is reduced (HR 1.07; CI 0.97–1.17). For men, the HR drops to 1.17 (CI 1.06–1.28) when co-variates are included (Model 3:2). Additional adjustment for previous psychiatric illness (Model 3:3) further reduces the estimate, leaving the HR for exposed men at 1.13 (CI 1.03–1.24).

**Table 4:** Distribution of individual and relational covariates used in the statistical analyses. The relational variables are intended to capture the ‘sociodemographic closeness’ between the cohort member and their sibling (applicable only for the exposed group).

	Eligible trials		Cohort members^a^		
	Exposed	Non-exposed	All	Women	Men
Women (%)	49.6	48.9	49.0		
Age at start of trial (mean)	57.5	57.3	52.1	52.1	52.1
Educational level (%)					
*Lower secondary or less*	21.1	19.5	20.2	14.8	25.4
*Upper secondary*	51.4	52.1	52.1	51.7	52.5
*Tertiary*	27.5	28.4	27.7	33.5	22.2
Married/reg. partner (%)	58.4	58.4	57.7	59.6	56
Previous psychiatric diagnosis (%)	5.5	4.7	2.7	2.7	2.7
**n**	**35 781**	**8 697 699**	**80 281**	**39 355**	**40 926**
**Relational variables**					
Sex composition (%)					
*CM*:*brother & S*:*brother*	21.6				
*CM*:*sister & S*:*sister*	28.4				
*CM*:*brother & S*:*sister*	28.9				
*CM*:*sister & S*:*brother*	21.2				
Age difference (%)					
*Difference ≤ 2 years*	19.0				
*S >2 years older*	48.5				
*S >2 years younger*	32.5				
Educational differences (%)					
*Same educational level*	49.7				
*S higher education than CM*	25.5				
*CM higher education than S*	24.8				
Marital status differences (%)					
*CM*:*married & S*:*married*	36.4				
*CM*:*married & S*:*unmarried*	22.1				
*CM*:*unmarried & S*:*married*	22.4				
*CM*:*unmarried & S*:*unmarried*	19.1				
Closeness in place of residency (%)					
*Living in same municipality*	35.2				
*Less than 200 km*	40.0				
*200–499 km*	15.3				
*≥500 km*	9.5				
**n** ^**b**^	**34 457**				

CM = cohort member; S = sibling

^a^ Values at first eligible trial

^b^ Not including trials with missing values on sibling variables

**Table 5:** Hazard ratios for receiving a psychiatric diagnosis within two years, by exposure to sibling cancer. Adjusted for age, marital status, educational level, number of siblings^a^ and previous psychiatric diagnoses^b^.

	All			Women			Men		
	Model 1:1	Model 2:1	Model 3:1	Model 1:2	Model 2:2	Model 3:2	Model 1:3	Model 2:3	Model 3:3
	HR (95% CI)	HR (95% CI)	HR (95% CI)	HR (95% CI)	HR (95% CI)	HR (95% CI)	HR (95% CI)	HR (95% CI)	HR (95% CI)
Exposed to sibling cancer	1.15	1.14	1.10	1.11	1.11	1.07	1.19	1.17	1.13
	(1.08–1.23)	(1.07–1.22)	(1.03–1.17)	(1.01–1.22)	(1.01–1.22)	(0.97–1.17)	(1.09–1.31)	(1.06–1.28)	(1.03–1.24)
Age (cont.)	0.98	0.98	0.93	0.97	0.97	0.93	0.99	0.99	0.94
	(0.98–0.99)	(0.98–0.99)	(0.93–0.94)	(0.97–0.98)	(0.97–0.98)	(0.92–0.94)	(0.98–0.99)	(0.98–1.00)	(0.93–0.95)
Married/registered partnership		0.46	0.58		0.49	0.59		0.43	0.56
		(0.44–0.49)	(0.55–0.60)		(0.46–0.53)	(0.56–0.63)		(0.40–0.46)	(0.52–0.60)
Not married/registered		1.00	1.00		1.00	1.00		1.00	1.00
partnership (ref.)									
*Educational level*:									
Lower secondary or less		1.32	1.22		1.31	1.21		1.40	1.26
		(1.23–1.42)	(1.14–1.30)		(1.17–1.46)	(1.10–1.33)		(1.25–1.56)	(1.14–1.39)
Upper secondary		1.15	1.11		1.11	1.08		1.23	1.15
		(1.08–1.22)	(1.05–1.17)		(1.03–1.21)	(1.01–1.16)		(1.11–1.36)	(1.06–1.26)
Tertiary (ref.)		1.00	1.00		1.00	1.00		1.00	1.00
Number of siblings^1^		1.01	1.01		0.99	0.99		1.03	1.02
		(0.99–1.03)	(0.99–1.03)		(0.963–1.020)	(0.96–1.02)		(1.00–1.06)	(1.00–1.05)
Previous psychiatric diagnosis			11.73			10.67			12.79
			(11.12–12.37)			(9.89–11.52)			(11.88–13.78)
Observations	8,733,480	8,733,480	8,733,480	4,275,052	4,275,052	4,275,052	4,458,428	4,458,428	4,458,428

^a^ Alive and living in Sweden at the start of trial.

^b^ Any previous psychiatric diagnoses registered after 1996.

95% confidence intervals, estimated on standard errors adjusted for trials clustered on individuals.

Models 1:1, 1:2 and 1:3 are adjusted for age. Models 2:1, 2:2 and 2:3 are adjusted for age, marital status, educational level and number of siblings. Models 3:1, 3:2 and 3:3 are adjusted for age, marital status, educational level, umber of siblings and previous psychiatric diagnoses.

**Fig 2:**
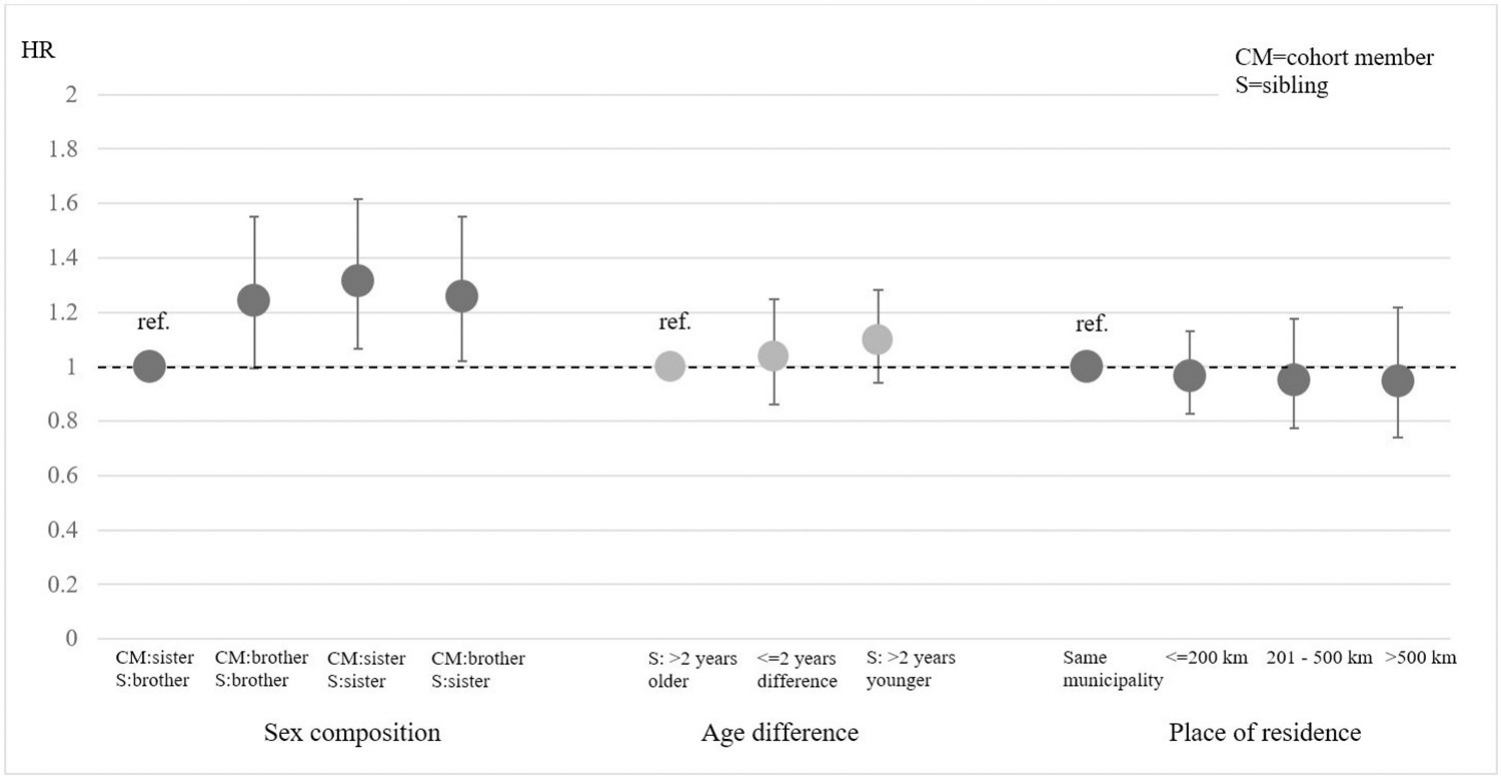
Hazard ratio for receiving a psychiatric diagnosis within 2 years from a sibling’s cancer diagnosis. Sociodemographic closeness by sex, age and place of residence. Exposed group, n = 34 457.

**Fig 3:**
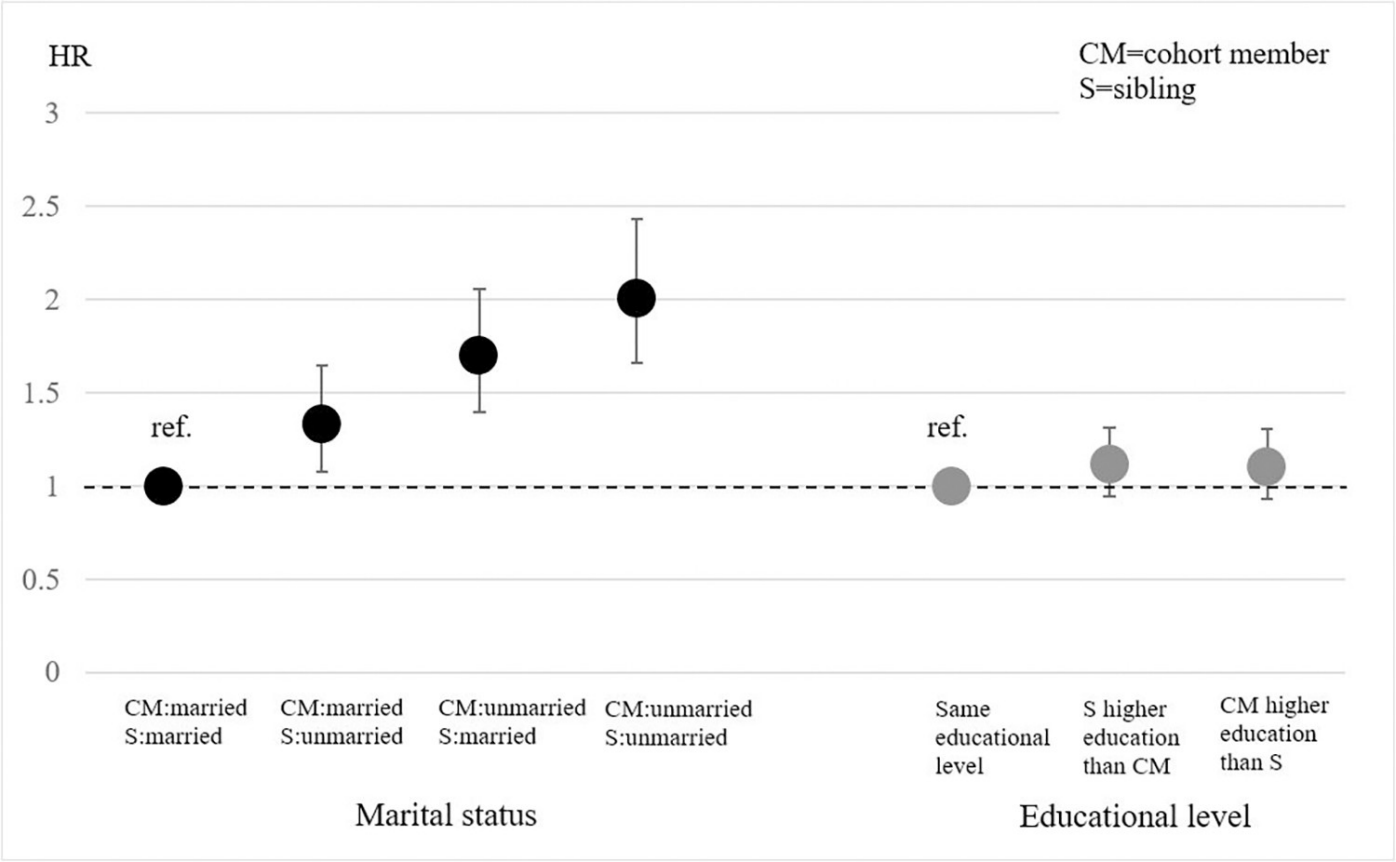
Hazard ratio for receiving a psychiatric diagnosis within 2 years from a sibling’s cancer diagnosis. Sociodemographic closeness by marital status and educational level. Exposed group, n = 34 457.

To investigate our second hypothesis, we performed sub-analyses of the exposed group (n = 34 457) according to the ‘sociodemographic closeness’ between the siblings in terms of sex, age, residency, marital status, and educational level. Results are presented in Figs 2 and 3.

Regarding the sex composition of the sibling-pair, women with a cancer-stricken sister run the highest risk of subsequent psychiatric illness (HR 1.31; CI 1.07–1.61) compared to women with a cancer-stricken brother (Fig 2). The risk for male cohort members is higher compared to the reference group, regardless of whether the sibling is a brother (HR 1.24; CI 0.99–1.55) or a sister (HR 1.26; CI 1.02–1.56). However, our findings do not support the idea of a higher risk of psychiatric illness following a sibling’s cancer diagnosis when siblings are close in age or residency.

For marital status, we find groups characterized by ‘closeness’ at both the highest and the lowest risk (Fig 3). The lowest HR is found for married cohort members whose cancer-stricken sibling is likewise married, while the highest HR is displayed when both siblings are unmarried (HR 2.01; CI 1.65–2.43). In sibling-pairs where one is married and the other is unmarried, the risk is intermediate, but with a higher HR where the cohort member is unmarried (HR 1.70; CI 1.40–2.06) than when the sibling is the unmarried one (HR = 1.33; CI 1.08–1.65). Finally, our results do not support the idea that exposure to a sibling’s cancer diagnosis is associated with a higher risk of psychiatric when the siblings are more similar in their educational level.

## Discussion

In this study, we investigated two hypotheses. Firstly, that a sibling’s cancer diagnosis is predictive of an increased risk for psychiatric illness in the other sibling. Secondly, that this ‘spill-over’ effect is higher in cases where the sociodemographic closeness between siblings is high. We emulated a target trial of the effect of one sibling’s cancer diagnosis on the other sibling’s risk of psychiatric diagnosis within the following 24 months, using observational data with medical diagnoses for a Swedish total-population cohort born in 1953, and their full biological siblings.

Our results support the first hypothesis, namely that individuals who are exposed to a sibling’s cancer diagnosis have a small, but robust, excess risk for subsequent psychiatric illness when relevant co-variates have been taken into consideration. Sex-specific analyses also revealed that the hypothesis held true for both genders, but that the association was stronger for men than for women. According to theories about linked lives, individuals with whom one has formed strong social bonds continue to be important sources of social, emotional, and practical attachment throughout the life-course [1, 8]. The present study shows that these attachments can affect individuals also in cases of adverse life events, such as a sibling’s cancer diagnosis. Our findings are in line with both the principals of linked lives [1] and with research performed within the field of bereavement [10]. Previous research also suggest that sibling relationships vary in intensity over the life course, but that they tend to gain an increased prominence in older ages [9]. In this study, we investigated the ‘spill-over’ effect for cohort members aged 52–66 years, spanning from post-family-formation to retirement age. The reported effects could, thus, be expected to differ in strength during other parts of the life-course. Adverse events in a sibling’s life can trigger underlying vulnerabilities such as previous psychiatric illness, and the findings of our study highlight that it may be important for the health care system to include siblings in the family-perspective of adult patients [23]. However, the general results remained also when previous psychiatric diagnoses were adjusted for. That is, even among individuals without a previous history of psychiatric illness, the exposure to a sibling’s cancer diagnosis can be a risk factor for psychiatric ill health.

Overall, our results provided only partial support for the second hypothesis, i.e. that sociodemographic closeness between siblings increases the risk of this type of ‘spill-over’ effects. Neither for age, place of residence, nor for educational level could any such pattern be detected. However, for sex and marital status, some support was found. Closeness in terms of biological sex increased the ‘spill-over’ effect for women, but no corresponding increase was found for men. Furthermore, the largest ‘spill-over’ effect was found among unmarried sibling-pairs, while the lowest effect was found among siblings who were both married. The finding for female sibling-pairs aligns with our second hypothesis and is consistent with previous research highlighting the strong bond between sisters [15, 17, 24]. However, the result for male cohort members is less consistent with cohesion among brothers [14, 16] since the ‘spill-over’ effect for men was equally strong regardless of the sex of the cancer-stricken sibling. Concerning the marital status composition of the sibling-pairs, our results align with the second hypothesis and previous suggestions of siblings serving as especially important sources of support and emotional closeness for unmarried individuals [9, 23]. Past research has also shown that sibling-pairs in middle and late adulthood, who are single or widowed, tend to report a closer relationship with each other [25]. Thus, unmarried persons may experience greater emotional distress than married individuals when a sibling is afflicted with cancer, due to the fear of losing their supportive network.

In essence, our results indicate that the intricacies of siblings’ linked lives, and their emotional closeness and support over the life course, can be partially explained in terms of ‘sociodemographic closeness’. However, it should be noted that we rely on proxy measures assumed to be correlated with the emotional bond between siblings, as no self-reported information available on this aspect.

When approaching this study, our greatest challenge was to define a reference group of non-exposed cohort members that could be followed up during the corresponding time-window as the exposed group. Here, the use of emulated clinical trials (ECT) provided an elegant solution. To the best of our knowledge, ECT has not previously been used for social scientific research questions. Randomized control trials are rarely appropriate in social science and this study demonstrates that the framework of ECT can be a fruitful way to approach questions of causality also in social scientific research.

Still, the use of register data to estimate causal effects comes with some limitations. Firstly, assignment into the treatment group assumed that the cohort members were aware of their siblings’ cancer diagnoses, an assumption that is likely violated for an unknown number of cohort members. Secondly, our data does not discriminate between sibling cancer diagnoses referring to suspected or confirmed cancers, nor does it account for the cancer stage, treatment groups or death if appropriate. However, since the causal contrast for our target trial is ‘intention-to-treat’, this is analogous to not knowing whether a patient adheres to the prescribed treatment. Nevertheless, it makes us unable to emulate the ‘per protocol effect’. Thirdly, information about municipality of residency was only available until December 2015, which most likely caused some misspecification of the sibling-pairs’ place of residence and, thus, their geographical closeness. However, results from sensitivity analyses that excluded trials starting from January 2016 and from January 2014 were largely consistent with the findings presented in this study (**S1 and S2 Tables**). Furthermore, no information was available about family history of psychiatric diagnosis; adding this in future studies would allow further understanding of the underlying mechanisms and vulnerabilities explaining the observed relationships.

In summary, this study demonstrated the existence of a ‘spill-over’ effect from one sibling’s negative life event, measured as a cancer diagnosis, to the psychiatric condition of the other sibling in a middle-aged Swedish total-population cohort born in 1953. The results partially aligned with the hypothesis that this ‘spill-over’ is amplified when the siblings are ‘close’ to each other in terms of sociodemographic characteristics. Thus, female and unmarried sibling-pairs exhibited stronger spill-over effects compared to their respective reference categories.

Our findings are in line with theories of linked lives, suggesting that relationships with members of the family of origin continue to impact people’s wellbeing throughout life [1, 2]. The study also adds to the literature on linked lives by showing that middle-aged siblings tend to share the emotional burden when disease strikes one of them.

## Supporting information

S1 Table Sensitivity analyses, for analyses presented in Table 4. (DOCX)

S2 Table Sensitivity analysis for results presented in Figs 2 and 3. Exposed group. Age adjusted hazard ratios.
(DOCX)
