# Supplementary material for: Using emulated clinical trials to investigate the risk of being diagnosed with psychiatric ill health following the cancer diagnosis of a sibling
Source: PLoS One. 2024 Apr 18;19(4):e0298175. doi: 10.1371/journal.pone.0298175 (PMC11025746; doi:10.1371/journal.pone.0298175)
Supplement: S1 Table — (DOCX) [file pone.0298175.s001.docx]

| **Supplementary Table S1.** Sensitivity analyses, for analyses presented in Table 4. | | | | | | |
| --- | --- | --- | --- | --- | --- | --- |
|  | **Trials starting 2005 – 2015^a^** | | | **Trials starting 2005 – 2013^b^** | | |
|  | **All** | **Women** | **Men** | **All** | **Women** | **Men** |
| Age adjusted hazard ratio for the exposed group (Model 1). | 1.18 | 1.13 | 1.22 | 1.18 | 1.18 | 1.18 |
| (95 % Confidence intervals) | (1.10 - 1.26) | (1.02 - 1.25) | (1.11 - 1.35) | (1.09 - 1.27) | (1.06 - 1.31) | (1.06 - 1.32) |
| Hazard ratio for the exposed group, fully adjusted (Model 3). | 1.12 | 1.09 | 1.15 | 1.13 | 1.13 | 1.14 |
| (95 % Confidence intervals) | (1.04 - 1.20) | (0.98 - 1.20) | (1.05 - 1.27) | (1.05 - 1.22) | (1.01 - 1.25) | (1.02 - 1.27) |
| Observations | 7,781,551 | 3,813,889 | 3,967,662 | 6,672,071 | 3,268,868 | 3,403,203 |

^a^ Full information on municipality of residency for both cohort member and siblings when assigning eligibility

^b^ Full information on municipality of residency for the cohort member until end of follow-up
