# Supplementary material for: Using emulated clinical trials to investigate the risk of being diagnosed with psychiatric ill health following the cancer diagnosis of a sibling
Source: PLoS One. 2024 Apr 18;19(4):e0298175. doi: 10.1371/journal.pone.0298175 (PMC11025746; doi:10.1371/journal.pone.0298175)
Supplement: S2 Table — Exposed group. Age adjusted hazard ratios. (DOCX) [file pone.0298175.s002.docx]

| **Supplementary Table S2.** Sensitivity analysis for results presented in Figures 2 and 3. Exposed group. Age adjusted hazard ratios. | | |
| --- | --- | --- |
|  | **Trials starting 2005 – 2015^a^** | **Trials starting 2005 – 2013^b^** |
|  | Hazard ratio (95% confidence intervals) | Hazard ratio (95% confidence intervals) |
| Sex |  |  |
| CM sister & S sister | ref | ref |
| CM brother & S brother | 0.94 (0.77 - 1.15) | 0.84 (0.68 - 1.05) |
| CM brother & S sister | 0.95 (0.79 - 1.13) | 0.91 (0.75 - 1.10) |
| CM sister & S brother | 0.74 (0.59 - 0.92) | 0.79 (0.63 - 0.99) |
| Age |  |  |
| S > 2 years older than CM | ref | ref |
| <= 2 years age difference | 1.04 (0.86 - 1.26) | 1.04 (0.84 - 1.27) |
| S > 2 years younger than CM | 1.10 (0.94 - 1.30) | 1.08 (0.90 - 1.28) |
| Marital status |  |  |
| CM married & S married | ref | ref |
| CM married & S unmarried | 1.26 (1.00 - 1.57) | 1.21 (0.95 - 1.54) |
| CM unmarried & S married | 1.97 (1.62 - 2.41) | 1.99 (1.61 - 2.46) |
| CM unmarried & S unmarried | 2.49 (2.05 - 3.06) | 2.39 (1.94 - 2.94) |
| Educational level |  |  |
| S same educational level as CM | ref | ref |
| S higher education than CM | 1.14 (0.96 - 1.35) | 1.15 (0.95 - 1.38) |
| CM higher education than S | 1.09 (0.91 - 1.30) | 1.08 (0.90 - 1.31) |
| Place of residence |  |  |
| Same municipality | ref | ref |
| <=200 km | 0.95 (0.81 - 1.12) | 0.89 (0.75 - 1.06) |
| 201-500 km | 0.93 (0.74 - 1.16) | 0.95 (0.75 - 1.19) |
| >500 km | 0.97 (0.75 - 1.25) | 1.06 (0.81 - 1.38) |
| Observations | 30,382 | 25,820 |
| ^a^ Full information on municipality of residency for both cohort member and siblings when assigning eligibility  ^b^ Full information on municipality of residency for the cohort member until end of follow-up  CM = Cohort member, S = Sibling | | |
